# Closing the loop – The human role in artificial intelligence for education

**DOI:** 10.3389/fpsyg.2022.956798

**Published:** 2022-08-25

**Authors:** Manuel Ninaus, Michael Sailer

**Affiliations:** ^1^Institute of Psychology, University of Graz, Graz, Austria; ^2^LEAD Graduate School and Research Network, University of Tübingen, Tübingen, Germany; ^3^Department of Psychology, Ludwig-Maximilians-Universität München, Munich, Germany

**Keywords:** technology enhanced learning, artificial intelligence (AI), machine learning (ML), adaptivity, digital technologies, education

## Abstract

Recent advancements in artificial intelligence make its use in education more likely. In fact, existing learning systems already utilize it for supporting students’ learning or teachers’ judgments. In this perspective article, we want to elaborate on the role of humans in making decisions in the design and implementation process of artificial intelligence in education. Therefore, we propose that an artificial intelligence-supported system in education can be considered a closed-loop system, which includes the steps of (i) data recording, (ii) pattern detection, and (iii) adaptivity. Besides the design process, we also consider the crucial role of the users in terms of decisions in educational contexts: While some implementations of artificial intelligence might make decisions on their own, we specifically highlight the high potential of striving for hybrid solutions in which different users, namely learners or teachers, are provided with information from artificial intelligence transparently for their own decisions. In light of the non-perfect accuracy of decisions of both artificial intelligence-based systems and users, we argue for balancing the process of human- and AI-driven decisions and mutual monitoring of these decisions. Accordingly, the decision-making process can be improved by taking both sides into account. Further, we emphasize the importance of contextualizing decisions. Potential erroneous decisions by either machines or humans can have very different consequences. In conclusion, humans have a crucial role at many stages in the process of designing and using artificial intelligence for education.

## Introduction

Imagine participating in an online course hosted on an automated AI-supported learning management system (LMS). After you have completed the latest chapter, the LMS points out that you failed to understand a specific issue in the learning material. Consequently, the system automatically repeats the latest course section you had already studied. Critically, the judgment of the system is wrong. Such a situation might demotivate you to continue with the course, or you might have lost your trust in the system. The AI-supported LMS drew wrong conclusions based on the available data about you and your learning process, recognized an incorrect pattern in your data, and failed to adapt the system to your actual needs.

**FIGURE 1:**
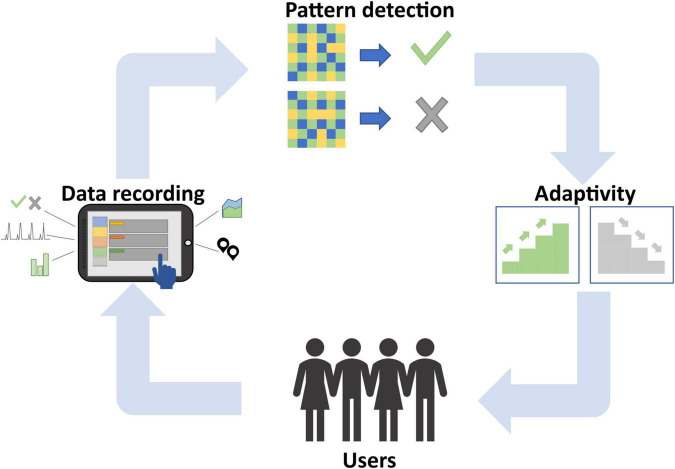
Closed-loop system for AI in education, including the steps (i) data recording, (ii) pattern detection, and (iii) adaptivity.

With this simplified example of a learning situation in digital learning environments, we want to illustrate that AI-based systems typically do not have 100% accuracy in their judgment. This might lead to devastating results on the learners’ or the teachers’ end. In the current article, we want to emphasize that the accuracy of predictions of AI-based systems depends on several steps that make up such a system and that humans can and should play a critical role as decision-makers along those steps and along the learning process. Specifically, we argue that AI-supported learning systems can be described as a closed-loop system (see Figure 1) as we know it from other feedback-rich learning systems such as neurofeedback (e.g., Ninaus et al., 2013), brain-computer interfaces (e.g., Liarokapis et al., 2014; Kober et al., 2018) and learning analytics systems (e.g., Clow, 2012). In particular, we suggest a closed-loop system for AI-supported learning systems, which consists of the following steps: (i) data recording, (ii) pattern detection, (iii) adaptivity. In the following, we will briefly highlight each of those steps with a particular emphasis on the critical role of humans.

## Data recording

Today’s hardware, network technologies, and data processing methods allow for the recording and processing of highly heterogeneous and multi-modal data (e.g., Di Mitri et al., 2018). Sensors can provide us not only with contextual data such as time, temperature, or location, but also with very personal data. The latter can be divided into behavioral (e.g., “clicks,” comments, time spent on a page) and physiological data (e.g., heart rate, electrodermal activity, brain activity). These data are particularly well suited for mapping processes because they can be recorded at a high sampling rate. Accordingly, the data can provide a (more) comprehensive picture of the learning process itself (for a review see Baker et al., 2020).

Nowadays, many people already use physiological sensors to track physical activity (for a review see, e.g., Gal et al., 2018). In contrast, the use of physiological and behavioral data to record and optimize learning activities is still rare in learning contexts, especially related to personalization of learning tasks in real-time. Undoubtedly, this will change in the future, as a growing number of studies show that physiological and behavioral data of learners are valuable for generating user models and fostering learning (for reviews see Mangaroska and Giannakos, 2019; Ninaus and Nebel, 2021). For instance, Li et al. (2020) used behavioral clickstream data from an LMS to predict performance in a course. Appel et al. (2021), on the other hand, used eye movement parameters to predict learners’ cognitive load in a game-based simulation. Compared to traditional performance data available after completing a learning task (e.g., scores, grades), continuously recorded physiological and behavioral data can provide deeper insight into cognitive, emotional, and motivational processes.

Even if the pure recording of data is automatic and thus purely machine-based, humans as decision-makers play a crucial role in (i) selecting appropriate sensors and metrics promising for the learning context, (ii) choosing data to be recorded, and (iii) implementing hardware and software architecture to record the data (see Di Mitri et al., 2018). In all of these steps, data handling has to be considered to be sustainable, responsible, and ethical (for a comprehensive discussion see Hakimi et al., 2021). This includes the transparency of data collection, appropriate communication with relevant stakeholders (see Drachsler and Greller, 2016), the use of established theoretically sound approaches for data selection, and the recording of data that indeed has the potential to foster learning. These aspects require expertise from a wide range of disciplines, such as computer science, psychology, and educational science as well as the collaboration between practitioners and researchers.

## Pattern detection

The selection of sensors and data to be recorded leads directly to the next step in our closed-loop system. Learning is a complex and dynamic process. Thus, it is unlikely to map and explain such a process using single data points, such as exam grades or a summative score. Accordingly, large amounts of data are necessary to better understand the learning process. However, as human perception and processing capacity cannot monitor numerous data sources simultaneously, interpretation of large amounts of data and metrics is difficult. Therefore, the focus of the next step in the loop is the identification of patterns in data using ML methods. Specifically, establishing a relationship between different parts of data (e.g., interaction duration with certain learning material) and a target variable (e.g., correct response).

For example, Brandl et al. (2021) recorded each click in a simulation for learning to diagnose patients with diseases. They were able to predict correct or incorrect diagnoses by using ML algorithms. The ML algorithm was used to identify activities that had the greatest influence on correct or incorrect diagnoses. In another study, automated facial emotion detection together with ML was used to classify whether individuals engaged in a game-based or a non-game-based mathematics learning task (Ninaus et al., 2019). Even though the prediction was successful, the used ML algorithm did not provide information on which emotions or magnitude thereof were relevant for successful prediction.

In both of these studies, ML was used to identify patterns in the recorded data. However, their approaches and interpretability of the results differed clearly. This can be partially attributed to the ML algorithm used (Random Forest Model vs. Support Vector Machine). The selection and decision for or against a particular ML algorithm is another key aspect in AI-supported learning systems, which should not only be data-driven but also informed by theory and determined by the overall goal.

Furthermore, differences between supervised and unsupervised ML algorithms should also be considered. The primary goal of supervised ML is to establish a relationship between different parts of the data (e.g., different activities in a simulation) and a target variable (e.g., correct/incorrect response; see Brandl et al., 2021). In unsupervised ML methods, the focus is on exploratory data analysis and clustering of data. Typically, there is no specific outcome variable, such as study success. Instead, one of the aims is to identify subgroups from a set of existing data which can be used for further analysis (e.g., Huijsmans et al., 2020).

However, as mentioned above, learning is a complex and dynamic process. Thus, learning processes might not be simple enough to be represented in a model that humans can always understand (for a comprehensive discussion, see Yarkoni and Westfall, 2017). For instance, ML and AI could be used to predict dropout rates in college or learning success for a course, but the underlying mechanisms might remain hidden from us. Nevertheless, the recent trend toward interpretable ML addresses the criticism of conventional ML of merely providing predictions and emphasizes transparency of the inner workings of ML models to better understand ML-guided decision-making (for a deeper methodological discussion, see Hilbert et al., 2021). This is especially relevant when studying learning processes, as it is crucial to find out which individual variables or aspects of an intervention positively or negatively influence learning success. This information can inform and influence the adaptation of a digital learning environment.

## Adaptivity

The next step in our proposed loop concerns the question of how the automatically detected pattern can be used in a learning environment to foster learning. One option is to directly provide detected information to different stakeholders involved in the process: learners and teachers. Learners can receive information about the detected sequences or patterns as feedback on the current performance. This information might be further processed in digital learning environments and provide learners with suggestions on how to adapt to certain problems that might have occurred in their learning process (see Plass and Pawar, 2020).

Similarly, teachers can also receive information about detected sequences and patterns of the learners’ learning process. This can help them improve their judgments based on the information received and eventually initiate support. One way is the use of teacher dashboards, which provide teachers with elaborated information about students’ learning processes. Further, teacher dashboards can automatically suggest support measures for specific learners (see Wiedbusch et al., 2021).

While in the two examples above, learners or teachers are responsible for making decisions, a third option is to leave the decision about adapting the learning environment to the learning environment itself. The idea of this approach of adaptivity in learning contexts is to provide learners with the exact learning experience and support that learners need in a particular situation to successfully achieve intended learning goals (Plass and Pawar, 2020).

By adapting learning environments and the therein contained support structures to the learners’ needs, personalized learning becomes possible (Bernacki et al., 2021). Reviews show that personalized learning in adaptive learning environments can have a positive impact on student learning (see Aleven et al., 2016; Bernacki et al., 2021; Ninaus and Nebel, 2021). However, more specific questions, such as which aspects of learning environments and according to which variables should be adapted to in order to foster learning remains largely unresolved.

Regarding adjustments of learning environments, macro-level and micro-level adaptivity can be distinguished (Plass and Pawar, 2020). On the one hand, macro-level adaptivity refers to adjustments regarding general categories of the wider learning context like the provision of suggestions for suitable learning material or courses based on the aggregation of events in learning environments (Sevarac et al., 2012; Mah and Ifenthaler, 2018). On the other hand, micro-level adaptivity focuses on currently processed learning tasks and thus on adapting the learning environment to the learner’s needs just-in-time (Plass and Pawar, 2020). If we consider the question of how micro-adaptivity can be established in learning environments, feedback approaches (Hattie and Timperley, 2007) and scaffolding approaches (Belland et al., 2017) stand out.

Especially for complex learning tasks, providing feedback on process or self-regulation level is necessary to master the necessary steps for solving a problem or to effectively monitor task performance (Wisniewski et al., 2020). Adaptive feedback might be especially promising on process or self-regulation level to develop an understanding of the current state of knowledge and identify the differences to an optimal state of knowledge. Further, adaptive feedback can feed back flawed task processing just in time (Narciss et al., 2014; Bimba et al., 2017). While some of this ideas have been tested in the context of intelligent tutoring systems, which are based on logfiles and closed-end questions (Graesser et al., 2018), AI-based methods can also provide a merit when complex tasks require students to write open text answers. AI-based approaches like Natural Language Processing (Manning and Schütze, 2005) can automatically analyze written text and allow for adaptively activating different feedback elements or different solutions based on the students’ answers (Zhu et al., 2017, 2020; Sailer et al., 2022).

Besides adaptive feedback, different forms of adaptive scaffolding are promising in the context of AI. The basic idea of scaffolding is to support learners in their problem solving, thus promoting their acquisition of knowledge and skills (Belland et al., 2017). As the need for support can vary between and within learners during task processing, the idea of adaptive scaffolding is to provide students with the support they need in specific situations at a specific time (Radkowitsch et al., 2021). Cognitive, meta-cognitive (see Belland et al., 2017), socio-cognitive (see Radkowitsch et al., 2020), and affective-motivational scaffolds (see Schrader and Bastiaens, 2012) can profit from the use of AI as they can be precisely faded in or out depending on learners needs. However, also other types of adaptive scaffolds that address the complexity of the learning environment or the salience of particular aspects of a learning environment or a learning task might profit from the use of AI. This form of indirect support can be referred to as representational scaffolding (Fischer et al., 2022). Representational scaffolds can be used to systematically vary the complexity of the learning environment and the salience of its aspects relevant to learning (Stadler et al., 2019b; Chernikova et al., 2020) in order to enable learners to solve problems according to their respective levels of knowledge and skills (e.g., Stadler et al., 2019a).

## Closing the loop

As highlighted above, AI-supported learning systems rely on decisions made in several steps along the proposed loop (see Figure 1). In a nutshell, user data is recorded, from which relevant data can be pre-selected using theoretical (human decision) as well as data-driven (machine) selection processes. In a next step, relevant patterns in data are detected by specifically selected ML algorithms. Based on successful pattern detection, suggestions regarding adaptations of the learning environment to the learners needs are provided to teachers or learners or decisions about adaptations are directly executed by the system. Finally, the result of this personalization affects the users’ learning process, which will be reflected in the data. This new user data can be used to refine the overall process, for instance, by identifying patterns that indicate potential improvements of the user and their learning process, which in turn will affect personalization procedures.

The proposed closed-loop highlights the complexity of AI-supported learning systems. Some of the manifold decisions described in the different steps can be automated using digital technologies and AI. There is also evidence that users prefer judgments from algorithms instead of judgments of people, despite blindness to the algorithm’s process (Logg et al., 2019). However, in many respects, human decisions are essential in the process (see Baker, 2016; Ritter et al., 2016; Holstein et al., 2017, 2020) and require expertise and perspectives from various disciplines (e.g., Sailer et al., 2022). In this perspective article, we want to emphasize the crucial role of human decisions in the design and implementation process of AI in education. Accordingly, we suggest striving for hybrid solutions by balancing the process of human- and AI-driven decisions and mutual monitoring of these decisions, which is in line with current discussions and frameworks on AI use in education (see Holstein et al., 2020; Molenaar, 2021) and beyond such as medicine (e.g., for detecting tumors, Topol, 2019) and autonomous driving (Awad et al., 2018; Ning et al., 2022) where AI is already more established. In these latter domains, AI technology is still mainly used to support or assist humans but has not replaced them. In fact, intricate moral decisions (Awad et al., 2018) and discussions revolving around bias, transparency, privacy, and accuracy are at the center of AI applications in these domains (Topol, 2019), which will also increasingly accompany the use and implementation of AI in education (for a detailed discussion see Akgun and Greenhow, 2021). Furthermore, as learning is a highly complex process, we would argue that in education, we still have a very long way to go to utilize AI in a balanced way, and – similar to medicine and autonomous driving – hybrid solutions will be dominant. The boundaries between AI and human decision-making, however, will definitely fluctuate (see Molenaar, 2021).

In the context of education, we believe that AI will change or shape the responsibilities and tasks of the different stakeholders involved in the educational process (see Molenaar, 2021 for a more detailed description of the teachers’ role in hybrid human-AI systems), which might differ across learning domains, contexts, situations. Accordingly, we want to emphasize the critical role of human decisions in high stake situations. Let us think back, for instance, at the example in the beginning using the AI-supported LMS that drew the wrong conclusions and thus provided you with an incorrect adaptation. Let us add to this a situation with more serious consequences: It has been argued that AI-supported systems might be useful for grading (e.g., Rus et al., 2013; Timms, 2016; Chen et al., 2020), selection of promising candidates for a job (e.g., Black and van Esch, 2020), or even for healthcare decisions (e.g., Pakdemirli, 2019). In fact, AI-supported systems can be a massive support for all those circumstances, but we need to be aware that those systems are not 100% accurate but can commit errors.

We can contextualize these decisions or erroneous conclusions, for instance, within statistician hypothesis testing and differ between type I (e.g., the system classifies a pupil to be not ready for higher secondary education when they actually are) and type II errors (e.g., the system predicts someone to pass the class when indeed the person will fail). Type I or type II errors can have very different consequences, and accordingly, one has to decide on a case-by-case basis how much decision-making power is given to an AI. In most cases, a hybrid decision-making process will probably be most correct and fair. In particular, AI in education might be used to support decision making, i.e., basing the decision process on insights or even recommendations provided by the AI and your own experience, impressions, and conclusions (Ritter et al., 2016; Holstein et al., 2020). While neither the AI nor the humans involved will always make correct decisions, the decision-making process can be improved by taking both sides into account. For instance, when an AI comes to the same conclusions as a teacher, correct conclusions are more likely. In contrast, disagreements between AI and the teacher might shed led on potential erroneous conclusions that otherwise would have remained hidden. We hope that by showing the steps of an AI-supported system, we demonstrated that humans can have a crucial role at many stages in this process and that we can use AI to support our capacities.

## Data availability statement

The original contributions presented in the study are included in the article/supplementary material, further inquiries can be directed to the corresponding author/s.

## Author contributions

Both authors have an equal contribution during the process of conceptualizing and writing this perspective article. Both authors approved the submitted version.

## References

[B1] Akgun S. Greenhow C. (2021). Artificial intelligence in education: addressing ethical challenges in K-12 settings. *AI Ethics* [Epub ahead of print]. 10.1007/s43681-021-00096-7 34790956 PMC8455229

[B2] Aleven V. McLaughlin E. A. Glenn R. A. Koedinger K. R. (2016). “Instruction based on adaptive learning technologies,” in *Handbook of research on learning and instruction*, eds Mayer R. E. Alexander P. A. (Abingdon: Routledge), 522–560. 10.4324/9781315736419.ch24

[B3] Appel T. Gerjets P. Hoffman S. Moeller K. Ninaus M. Scharinger C. (2021). Cross-task and cross-participant classification of cognitive load in an emergency simulation game. *IEEE Trans. Affective Comput*. 10.1109/TAFFC.2021.3098237 [Epub ahead of print].

[B4] Awad E. Dsouza S. Kim R. Schulz J. Henrich J. Shariff A. (2018). The moral machine experiment. *Nature* 563 59–64. 10.1038/s41586-018-0637-6 30356211

[B5] Baker R. Xu D. Park J. Yu R. Li Q. Cung B. (2020). The benefits and caveats of using clickstream data to understand student self-regulatory behaviors: opening the black box of learning processes. *Int. J. Educ. Technol. High Educ.* 17:13. 10.1186/s41239-020-00187-1

[B6] Baker R. S. (2016). Stupid tutoring systems, intelligent humans. *Int. J. Artif. Intell. Educ.* 26 600–614. 10.1007/s40593-016-0105-0

[B7] Belland B. R. Walker A. E. Kim N. J. Lefler M. (2017). Synthesizing results from empirical research on computer-based scaffolding in STEM education: a meta-analysis. *Rev. Educ. Res.* 87 309–344. 10.3102/0034654316670999 28344365 PMC5347356

[B8] Bernacki M. L. Greene M. J. Lobczowski N. G. (2021). A systematic review of research on personalized learning: personalized by whom, to what, how, and for what purpose(s)? *Educ. Psychol. Rev.* 33 1675–1715. 10.1007/s10648-021-09615-8

[B9] Bimba A. T. Idris N. Al-Hunaiyyan A. Mahmud R. B. Shuib N. L. B. M. (2017). Adaptive feedback in computer-based learning environments: a review. *Adapt. Behav.* 25 217–234. 10.1177/1059712317727590

[B10] Black J. S. van Esch P. (2020). AI-enabled recruiting: What is it and how should a manager use it? *Bus. Horizons* 63 215–226. 10.1016/j.bushor.2019.12.001

[B11] Brandl L. Richters C. Radkowitsch A. Fischer M. R. Schmidmaier R. Fischer F. (2021). Simulation-based learning of complex skills: predicting performance with theoretically derived process features. *Psychol. Test Assess. Model.* 63 542–560.

[B12] Chen L. Chen P. Lin Z. (2020). Artificial intelligence in education: a review. *IEEE Access* 8 75264–75278. 10.1109/ACCESS.2020.2988510

[B13] Chernikova O. Heitzmann N. Fink M. C. Timothy V. Seidel T. Fischer F. (2020). Facilitating diagnostic competences in higher education—a meta-analysis in medical and teacher education. *Educ. Psychol. Rev.* 32 157–196. 10.1007/s10648-019-09492-2

[B14] Clow D. (2012). “The learning analytics cycle: closing the loop effectively,” in *Proceedings of the 2nd International Conference on Learning Analytics and Knowledge*, New York, NY.

[B15] Di Mitri D. Schneider J. Specht M. Drachsler H. (2018). From signals to knowledge: a conceptual model for multimodal learning analytics. *J. Comput. Assist. Learn.* 34 338–349. 10.1111/jcal.12288

[B16] Drachsler H. Greller W. (2016). “Privacy and Analytics – it’s a DELICATE Issue A Checklist for Trusted Learning Analytics,” in *Proceedings of the Sixth International Conference on Learning Analytics & Knowledge - LAK ’16*, New York, NY.

[B17] Fischer F. Bauer E. Seidel T. Schmidmaier R. Radkowitsch A. Neuhaus B. (2022). Representational scaffolding in digital simulations – learning professional practices in higher education. *PsyArXiv* [Preprint]. 10.31234/osf.io/bf92d

[B18] Gal R. May A. M. van Overmeeren E. J. Simons M. Monninkhof E. M. (2018). The effect of physical activity interventions comprising wearables and smartphone applications on physical activity: a systematic review and meta-analysis. *Sports Med. Open* 4:42. 10.1186/s40798-018-0157-9 30178072 PMC6120856

[B19] Graesser A. C. Hu X. Sottilare R. (2018). Intelligent tutoring systems. *Int. Handb. Learn. Sci.* 246–255. 10.4324/9781315617572

[B20] Hakimi L. Eynon R. Murphy V. A. (2021). The ethics of using digital trace data in education: a thematic review of the research landscape. *Rev. Educ. Res.* 91 671–717. 10.3102/00346543211020116

[B21] Hattie J. Timperley H. (2007). The power of feedback. *Rev. Educ. Res.* 77 81–112. 10.3102/003465430298487

[B22] Hilbert S. Coors S. Kraus E. Bischl B. Lindl A. Frei M. (2021). Machine learning for the educational sciences. *Rev. Educ.* 9:e3310. 10.1002/rev3.3310

[B23] Holstein K. Aleven V. Rummel N. (2020). “A Conceptual Framework for Human–AI Hybrid Adaptivity in Education,” in *Artificial Intelligence in Education Lecture Notes in Computer Science*, eds Bittencourt I. I. Cukurova M. Muldner K. Luckin R. Millán E. (Cham: Springer International Publishing), 240–254. 10.1007/978-3-030-52237-7_20

[B24] Holstein K. McLaren B. M. Aleven V. (2017). “Intelligent tutors as teachers’ aides: exploring teacher needs for real-time analytics in blended classrooms,” in *Proceedings of the Seventh International Learning Analytics & Knowledge Conference*, New York, NY.

[B25] Huijsmans M. D. E. Kleemans T. van der Ven S. H. G. Kroesbergen E. H. (2020). The relevance of subtyping children with mathematical learning disabilities. *Res. Dev. Disabil.* 104:103704. 10.1016/j.ridd.2020.103704 32574935

[B26] Kober S. E. Ninaus M. Friedrich E. V. C. Scherer R. (2018). “BCI and Games: Playful, Experience-Oriented Learning by Vivid Feedback?,” in *Brain–Computer Interfaces Handbook: Technological and Theoretical Advances*, eds Nam C. S. Nijholt A. Lotte F. (Boca Raton, FL: CRC Press - Taylor & Francis Group).

[B27] Li Q. Baker R. Warschauer M. (2020). Using clickstream data to measure, understand, and support self-regulated learning in online courses. *Internet High. Educ*. 45:100727. 10.1016/j.iheduc.2020.100727

[B28] Liarokapis F. Debattista K. Vourvopoulos A. Petridis P. Ene A. (2014). Comparing interaction techniques for serious games through brain-computer interfaces: a user perception evaluation study. *Entertain. Comput.* 5 391–399. 10.1016/j.entcom.2014.10.004

[B29] Logg J. M. Minson J. A. Moore D. A. (2019). Algorithm appreciation: people prefer algorithmic to human judgment. *Organ. Behav. Hum. Decis. Process.* 151 90–103. 10.1016/j.obhdp.2018.12.005

[B30] Mah D.-K. Ifenthaler D. (2018). Students’ perceptions toward academic competencies: the case of German first-year students. *Issues Educ. Res.* 28 120–137. 10.3316/informit.437867582603162

[B31] Mangaroska K. Giannakos M. (2019). Learning analytics for learning design: a systematic literature review of analytics-driven design to enhance learning. *IEEE Trans. Learn. Technol.* 12 516–534. 10.1109/TLT.2018.2868673

[B32] Manning C. Schütze H. (2005). *Foundations of Statistical Natural Language Processing*, 8th Edn. Cambridge, MA: MIT Press.

[B33] Molenaar I. (2021). *Personalisation of learning: Towards hybrid human-AI learning technologies.* Paris: OECD Publishing.

[B34] Narciss S. Sosnovsky S. Schnaubert L. Andrès E. Eichelmann A. Goguadze G. (2014). Exploring feedback and student characteristics relevant for personalizing feedback strategies. *Comput. Educ.* 71 56–76. 10.1016/j.compedu.2013.09.011

[B35] Ninaus M. Greipl S. Kiili K. Lindstedt A. Huber S. Klein E. (2019). Increased emotional engagement in game-based learning – A machine learning approach on facial emotion detection data. *Comput. Educ.* 142:103641. 10.1016/j.compedu.2019.103641

[B36] Ninaus M. Nebel S. (2021). A systematic literature review of analytics for adaptivity within educational video games. *Front. Educ.* 5:611072. 10.3389/feduc.2020.611072

[B37] Ninaus M. Witte M. Kober S. E. Friedrich E. V. C. Kurzmann J. Hartsuiker E. (2013). “Neurofeedback and Serious Games,” in *Psychology, Pedagogy, and Assessment in Serious Games*, eds Connolly T. M. Boyle E. Hainey T. Baxter G. Moreno-ger P. (Hershey, PA: IGI Global), 82–110. 10.4018/978-1-4666-4773-2.ch005

[B38] Ning H. Yin R. Ullah A. Shi F. (2022). A survey on hybrid human-artificial intelligence for autonomous driving. *IEEE Trans. Intell. Transport. Syst.* 23 6011–6026. 10.1109/TITS.2021.3074695

[B39] Pakdemirli E. (2019). Artificial intelligence in radiology: friend or foe? Where are we now and where are we heading? *Acta Radiol. Open* 8:205846011983022. 10.1177/2058460119830222 30815280 PMC6385326

[B40] Plass J. L. Pawar S. (2020). Toward a taxonomy of adaptivity for learning. *J. Res. Technol. Educ.* 52 275–300. 10.1080/15391523.2020.1719943

[B41] Radkowitsch A. Sailer M. Schmidmaier R. Fischer M. R. Fischer F. (2021). Learning to diagnose collaboratively – Effects of adaptive collaboration scripts in agent-based medical simulations. *Learn. Instr.* 75:101487. 10.1016/j.learninstruc.2021.101487

[B42] Radkowitsch A. Vogel F. Fischer F. (2020). Good for learning, bad for motivation? A meta-analysis on the effects of computer-supported collaboration scripts. *Intern. J. Comput. Support. Collab. Learn.* 15 5–47. 10.1007/s11412-020-09316-4

[B43] Ritter S. Yudelson M. Fancsali S. E. Berman S. R. (2016). “How Mastery Learning Works at Scale,” in *Proceedings of the Third (2016) ACM Conference on Learning @ Scale*, Edinburgh.

[B44] Rus V. D’Mello S. Hu X. Graesser A. (2013). Recent advances in conversational intelligent tutoring systems. *AIMag* 34 42–54. 10.1609/aimag.v34i3.2485

[B45] Sailer M. Bauer E. Hofmann R. Kiesewetter J. Glas J. Gurevych I. (2022). Adaptive feedback from artificial neural networks facilitates pre-service teachers’ diagnostic reasoning in simulation-based learning. *Learn. Instr.* [Preprint]. 10.1016/j.learninstruc.2022.101620

[B46] Schrader C. Bastiaens T. (2012). Learning in educational computer games for novices: the impact of support provision types on virtual presence, cognitive load, and learning outcomes. *IRRODL* 13:206. 10.19173/irrodl.v13i3.1166

[B47] Sevarac Z. Devedzic V. Jovanovic J. (2012). Adaptive neuro-fuzzy pedagogical recommender. *Expert Syst. Applic.* 39 9797–9806. 10.1016/j.eswa.2012.02.174

[B48] Stadler M. Niepel C. Greiff S. (2019b). Differentiating between static and complex problems: a theoretical framework and its empirical validation. *Intelligence* 72 1–12. 10.1016/j.intell.2018.11.003

[B49] Stadler M. Fischer F. Greiff S. (2019a). Taking a closer look: an exploratory analysis of successful and unsuccessful strategy use in complex problems. *Front. Psychol.* 10:777. 10.3389/fpsyg.2019.00777 31133910 PMC6514184

[B50] Timms M. J. (2016). Letting artificial intelligence in education out of the box: educational cobots and smart classrooms. *Int. J. Artif. Intell. Educ.* 26 701–712. 10.1007/s40593-016-0095-y

[B51] Topol E. J. (2019). High-performance medicine: the convergence of human and artificial intelligence. *Nat. Med.* 25 44–56. 10.1038/s41591-018-0300-7 30617339

[B52] Wiedbusch M. D. Kite V. Yang X. Park S. Chi M. Taub M. (2021). A theoretical and evidence-based conceptual design of metadash: an intelligent teacher dashboard to support teachers’ decision making and students’ self-regulated learning. *Front. Educ.* 6:570229. 10.3389/feduc.2021.570229

[B53] Wisniewski B. Zierer K. Hattie J. (2020). The power of feedback revisited: a meta-analysis of educational feedback research. *Front. Psychol.* 10:3087. 10.3389/fpsyg.2019.03087 32038429 PMC6987456

[B54] Yarkoni T. Westfall J. (2017). Choosing prediction over explanation in psychology: lessons from machine learning. *Perspect. Psychol. Sci.* 12 1100–1122. 10.1177/1745691617693393 28841086 PMC6603289

[B55] Zhu M. Lee H.-S. Wang T. Liu O. L. Belur V. Pallant A. (2017). Investigating the impact of automated feedback on students’ scientific argumentation. *Int. J. Sci. Educ.* 39 1648–1668. 10.1080/09500693.2017.1347303

[B56] Zhu M. Liu O. L. Lee H.-S. (2020). The effect of automated feedback on revision behavior and learning gains in formative assessment of scientific argument writing. *Comput. Educ.* 143:103668. 10.1016/j.compedu.2019.103668

